# Dissecting Digital Card Games to Yield Digital Biomarkers for the Assessment of Mild Cognitive Impairment: Methodological Approach and Exploratory Study

**DOI:** 10.2196/18359

**Published:** 2021-11-04

**Authors:** Karsten Gielis, Marie-Elena Vanden Abeele, Robin De Croon, Paul Dierick, Filipa Ferreira-Brito, Lies Van Assche, Katrien Verbert, Jos Tournoy, Vero Vanden Abeele

**Affiliations:** 1 e-Media Research Lab Katholieke Universiteit Leuven Leuven Belgium; 2 Memory Clinic Jessa Hospital Hasselt Belgium; 3 Department of Computer Science Katholieke Universiteit Leuven Leuven Belgium; 4 Department of Gerontopsychiatry University Psychiatric Center Duffel Belgium; 5 Instituto de Saúde Ambiental Faculdade de Medicina Universidade de Lisboa Lisboa Portugal; 6 Section of Geriatric Psychiatry University Hospital Leuven Katholieke Universiteit Leuven Leuven Belgium; 7 Department of Psychiatry University Hospital Leuven Katholieke Universiteit Leuven Leuven Belgium; 8 Department of Geriatric Medicine University Hospital Leuven Leuven Belgium; 9 Department of Public Health and Primary Care Gerontology and Geriatrics Katholieke Universiteit Leuven Leuven Belgium

**Keywords:** mild cognitive impairment, Klondike Solitaire, card games, generalized linear mixed effects analysis, expert study, monitoring, screening, cognition, dementia, older adults, mobile phone

## Abstract

**Background:**

Mild cognitive impairment (MCI), the intermediate cognitive status between normal cognitive decline and pathological decline, is an important clinical construct for signaling possible prodromes of dementia. However, this condition is underdiagnosed. To assist monitoring and screening, digital biomarkers derived from commercial off-the-shelf video games may be of interest. These games maintain player engagement over a longer period of time and support longitudinal measurements of cognitive performance.

**Objective:**

This paper aims to explore how the player actions of Klondike Solitaire relate to cognitive functions and to what extent the digital biomarkers derived from these player actions are indicative of MCI.

**Methods:**

First, 11 experts in the domain of cognitive impairments were asked to correlate 21 player actions to 11 cognitive functions. Expert agreement was verified through intraclass correlation, based on a 2-way, fully crossed design with type consistency. On the basis of these player actions, 23 potential digital biomarkers of performance for Klondike Solitaire were defined. Next, 23 healthy participants and 23 participants living with MCI were asked to play 3 rounds of Klondike Solitaire, which took 17 minutes on average to complete. A generalized linear mixed model analysis was conducted to explore the differences in digital biomarkers between the healthy participants and those living with MCI, while controlling for age, tablet experience, and Klondike Solitaire experience.

**Results:**

All intraclass correlations for player actions and cognitive functions scored higher than 0.75, indicating good to excellent reliability. Furthermore, all player actions had, according to the experts, at least one cognitive function that was on average moderately to strongly correlated to a cognitive function. Of the 23 potential digital biomarkers, 12 (52%) were revealed by the generalized linear mixed model analysis to have sizeable effects and significance levels. The analysis indicates sensitivity of the derived digital biomarkers to MCI.

**Conclusions:**

Commercial off-the-shelf games such as digital card games show potential as a complementary tool for screening and monitoring cognition.

**Trial Registration:**

ClinicalTrials.gov NCT02971124; https://clinicaltrials.gov/ct2/show/NCT02971124

## Introduction

### Assessing Cognitive Performance

Mild cognitive impairment (MCI) is a clinical entity defined as a transitional state between normal and pathological aging, where one or more cognitive domains are significantly impaired, yet activities of daily living are still preserved [1]. Early detection of MCI is important for signaling possible prodromes of dementia, monitoring the progression of possible decline, taking supportive measures, and detecting any possible underlying causes. However, cognitive impairment is still underdiagnosed [2-4]. In response, governmental bodies have called for novel, scalable, and longitudinal tools to assist in the early screening and monitoring of dementia [5-7]. To answer this call, researchers have explored the use of digital games as a suitable medium for assessing cognitive impairment [8-11]. Games are autotelic in nature, tapping into the intrinsic motivation to play [12,13], hence captivating a player’s attention. Furthermore, digital games are a natural source of information on player behavior, cognitive performance, motor skills, social conduct, and affective experiences [14].

As such, digital games may help by providing *digital* biomarkers of cognitive performance. Biomarkers, defined as “objectively measured and evaluated indicators of normal biological processes, pathogenic processes, or pharmacologic responses to a therapeutic intervention [15]” have a longstanding tradition in dementia research [16,17]. Complementary to their biological counterparts, *digital* biomarkers are “user-generated physiological and behavioral measures collected through connected *digital* devices to explain, influence and/or predict health-related outcomes [18].” User interaction with digital games produces dense and detailed behavioral traces that may inform on the users’ social health, praxis, and *cognition*.

Today, the focal point of research assessing cognitive performance is *serious games*, that is, games intentionally designed and built for a serious purpose and not solely to entertain [19]. Although serious gaming interventions show potential, they are typified by less funding, shorter development cycles, and missing know-how compared with traditional video games, which affect the in-game hallmarks of quality, such as graphics, music, and storytelling [20,21]. This may lead to frustrating player experiences, a lack of engagement, and less attention being paid during gameplay, which may lower the reliability and validity of any findings and possibly cause attrition in longitudinal studies [22-24]. Therefore, most recently, the study by Mandryk and Birk [14] argued in favor of turning to commercial off-the-shelf (COTS) video games instead [14]. Instead of spending limited resources on building a serious game, researchers can devote themselves to investigating existing games already enjoyed by the target population. Although not designed to measure cognition, COTS games are woven into the fabric of everyday life and may be able to provide digital biomarkers of cognitive performance that are reflective of cognitive status [14,25].

This study aims to explore the possibility of using COTS card games to screen cognition among patients living with MCI. This study involved 11 experts in the domain of MCI coming together to craft 23 candidate digital biomarkers from the digital card game Klondike Solitaire. Subsequently, a data acquisition campaign was set up involving 46 participants: 23 (50%) healthy older adults and 23 (50%) older adults with MCI. The participants were asked to play 3 games on a tablet. We examined the game data for differences at a group level for the candidate digital biomarkers using a generalized linear mixed model (GLMM) analysis. The results show that of the 23 candidate digital biomarkers, 12 (52%) differed significantly between both groups, taking age, tablet experience, and Klondike Solitaire experience into account. By providing a methodological approach and an exploratory study for crafting digital biomarkers, as well as articulating the rationale and the different steps taken, we hope to inform future researchers who aim to leverage the use of COTS video games to yield digital biomarkers.

### MCI Classification

Persons diagnosed with MCI show a deficit in cognition in at least one cognitive domain that cannot be attributed to age or any other disease; yet, they do not fulfill the diagnosis of dementia [26]. Persons with MCI, however, have a higher risk of progressing to a form of dementia such as Lewy body dementia [27], vascular dementia [28], or the most common form of dementia, Alzheimer disease [29]. Depending on the early symptoms, persons with MCI can be classified into 2 groups: amnestic MCI (aMCI) and nonamnestic MCI (naMCI). Persons in the aMCI group show a significant memory deficit, whereas in persons with naMCI, mainly a nonmemory impairment (eg, language) is present [30]. For both aMCI and naMCI, a further distinction can be made between persons with 1 cognitive domain impaired (single domain MCI) and those with multiple cognitive domains impaired (multiple-domain MCI). Although no treatments exist with the current state of modern medicine to cure the neuronal damage of these progressive forms of dementia [31,32], early diagnosis matters [33] because there are several measures that can be taken to slow down disease progression [34], including starting nonpharmacological treatment for delaying symptoms [31,32,34], or support patients and families with the appropriate counseling [35].

### Detecting MCI

Typically, the process leading to a diagnosis of MCI is set into motion by a cognitive complaint from the older adult, relative, or (informal) caregiver, followed by a presumptive identification through a screening test. The cognitive screening tests used most often to detect MCI are the Montreal Cognitive Assessment (MoCA) [36] and the Mini-Mental State Examination (MMSE) [37]. These cognitive screening tests primarily focus on evaluating language, visual skills, memory, orientation, attention, and executive functions [38]. Despite their widespread use, the psychometric properties of the screening tests by themselves are insufficient to draw firm conclusions regarding an MCI diagnosis [39].

Therefore, this presumptive identification is in turn followed by an elaborate neuropsychological assessment (ie, a cognitive test battery) and possibly a biomarker scan or a neuroimaging scan [26,30]. This neuropsychological assessment assesses cognitive skills and level of impairment more thoroughly. In addition, the assessment may include a semiguided interview with a relative or caregiver to evaluate the change in symptoms over time, such as in the Clinical Dementia Rating (CDR) Scale [40]. However, this neuropsychological assessment is laborious and time-intensive, requiring skilled test administrators, who, despite their training, are still subject to interassessor variability [41]. In addition, from a patient perspective, the process has been described as bewildering, highly stressful, and even humiliating [42,43], contributing to malperformance. This in turn can make patients self-aware of impairment, leading to feelings of distress or helplessness, possibly spiraling into even worse performance [44,45]. Although biological and imaging biomarkers are becoming more common to support diagnosis, they remain expensive and invasive, making them equally unfit for high-frequency measurements [41]. As a result, health professionals and policy makers welcome additional tools that support the monitoring of cognition [1,46-49] while reducing patient-level barriers and are more considerate of patients’ experiences [44].

### Serious Games for the Assessment of Cognitive Functions

Serious (digital) games are “games that do not have entertainment, enjoyment, or fun as their primary purpose [19].” An early and longstanding tradition [50] is the use of serious games for cognitive evaluation [51]. Space Fortress [19,50] is perhaps the first research game to measure and train cognitive control and related cognitive functions. Ever since, the popularity of creating serious games and game-based interventions to measure, detect, and train cognition has only increased, as indicated by systematic reviews on this topic by Ferreira-Brito et al [52], Lumsden et al [53], and Valladares-Rodriguez et al [51].

Serious games may provide certain advantages for the assessment of cognitive performance *compared with* standard cognitive assessment tests. First, by offering an interactive and immersive audiovisual experience, serious games can be considered to be more engaging than classical tests [23,51,53]. As ensuring the full attention of the participant is paramount in neuropsychological testing, such increased engagement may also result in more reliable research results; previous research has linked effort to cognitive test performance in healthy undergraduate students [54]. Second, games allow for embedding of cognitive tasks in a virtual, audiovisual world, which more closely mimics the actual lived-in world, allowing for better transfer of task results and providing *higher ecological validity* [55]. However, it has to be noted that the skills learned through serious games might still be difficult to generalize to the skills needed in a real-life context [55]. Third, serious games can be designed in such a manner that they minimize the need for the presence of a trained administrator. Setting a pace, reading out loud, or cueing can be integrated into the game itself. In this manner, test administration bias is reduced, and white-coat effects can be minimized [56,57]. If assessments are possible with less supervision and manual effort, they also become more *scalable* because testing becomes less resource intensive [55]*.* However, this lack of supervision while performing measurements has an important caveat. When measurements are performed in a personal environment, it becomes more difficult to prevent distractions that influence gameplay behavior.

Although serious games show promising results and have merit for both patient and physician, serious games are at risk of being dismissed as “chocolate-covered broccoli”: neuropsychological tests embellished with a thin layer of gameplay [58-60]. This can lead to games that are suboptimal in terms of esthetic quality and game mechanics [20], negatively affecting gameplay [58]. A meta-analysis of *serious games* [22] shows that although they can be more effective and improve retention compared with conventional methods, they are not found to be more motivating. Similar signs of lack of motivation have been noted in game-based interventions designed to train cognitive functions [23,24].

This lack of sustained engagement contrasts with surveys on gameplay among older adults. A large-scale (N=3737) survey of older adults’ attitude toward video games, conducted in 2019 by the American Association of Retired Persons [61], highlights that older adults enjoy playing digital games. Of the nine reasons to play, *to have fun* was indicated to be the top reason (78%) to play video games, with *to help stay mentally sharp* coming in second (69%). In the 70-years-or-older age category, this difference became marginal, with 73% indicating *to have fun* and 72% indicating *to stay mentally sharp* as the reasons to play. Therefore, to increase engagement and to tap into intrinsic motivation, popular COTS video games may present an interesting alternative. These games are already woven into the daily life of the older adult, providing *meaningful* play [62,63].

### COTS Video Games for Mental Health

COTS games may have the ability to retain players over a longer period and to support *continuous measurements of cognitive performance*. As frequent measurements are more sensitive to detecting small deviations in the cognitive performance of older adults [64], this could lead to a better interpretation of the patient’s cognitive trajectory. Furthermore, fluctuations in cognitive performance [65], a common feature of dementia, may be more easily detected. In addition, this continuous monitoring enables establishing an intraindividual cognitive baseline [66]. This cognitive baseline can be used to compare patients with themselves, as opposed to comparisons with normed references. In turn, this can lead to improved management and care [1]. Nevertheless, a prominent disadvantage of COTS games is that researchers have less control over which cognitive functions are measured in the game [60].

Recent studies on using COTS games to measure cognitive impairment have generated promising results. The study by Jimison et al [8] used FreeCell, a Solitaire variant, to compare cognitive performance between a group of people living with MCI and a healthy control group by means of an *optimized solver*. The results indicated that based on gameplay, the group with MCI could be discerned from the healthy control group [8]. In the case of sudoku, another popular game among older adults, the study by Grabbe [67] showed that performance in the game was significantly related to measures of working memory. Using a set of smartphone-based puzzle games, which also contained sudoku, the study by Thompson et al [68] explored smartphone-based games as a means of portable cognitive assessment and monitoring. The participants’ performance in these games correlated to several measures of cognition, among which were visual memory, verbal learning, and reasoning. Finally, the study by Wallace et al [11] developed a word search game and sudoku that incorporated hints to reduce frustration among patients living with MCI. Their first study with 2 patients indicated that cognitive performance could be measured with COTS gameplay, comparing game performance with the MoCA and the Repeatable Battery for the Assessment of Neuropsychological Status [69]. Synthesizing these results, these studies suggest that COTS games yield promise for the assessment of cognitive impairment but that further research is warranted.

Across the aforementioned studies, different lines of reasoning have been given to justify the game of choice as suitable for neuropsychological evaluation. The study by Grabbe [67] analyzed the components of sudoku and linked them to working memory based on a subjective analysis. The study by Jimison et al [8] chose FreeCell because it was the most popular game in their focus group. The study by Wallace et al [11] chose a word search game and sudoku above other games because of certain properties such as the percentage of successful deals. Finally, the study by Thompson et al [68] chose games based on face validity with regard to targeting cognitive functions. Although these reasons are valid arguments for choosing a game, it should be noted that these studies have no arguments rooted in empirical evidence for their game of choice.

### Klondike Solitaire

One of the most popular card games among older adults is Klondike Solitaire, also known as Patience, Fascination, or even just Solitaire [70]. The popularity of Klondike Solitaire among older adult gamers was recently noted in the study by Boot et al [71]. For 1 year, participants had access to a computer where 11 games were installed, among which were sudoku, Klondike Solitaire, and crossword puzzles. The study noted that “There was a strong, clear preference for Solitaire […]. After Solitaire, there was no clear second choice, and on average participants infrequently played the other games.” In addition, the results showed that of all 11 games, 1 (9%)—Solitaire—was being played most consistently.

**Figure 1:**
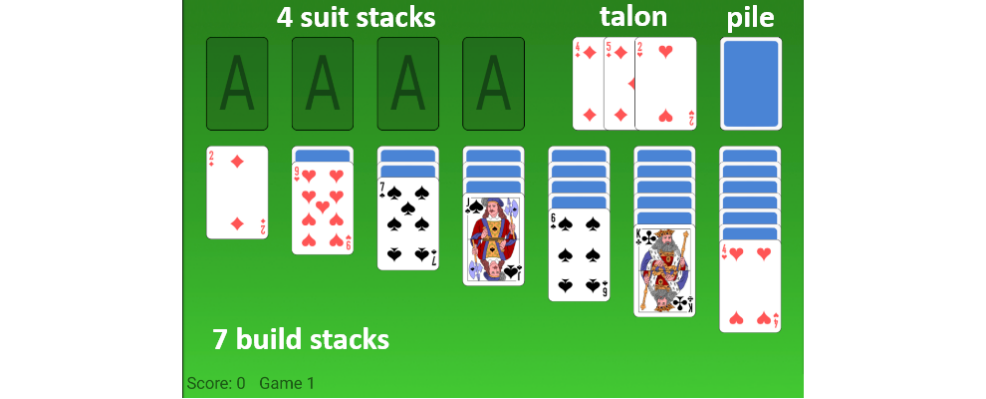
Klondike Solitaire. The seven build stacks can be seen at the bottom, the suit stacks are at the top left. The pile of undealt cards can be seen in the top right.

This popular card game is played with a standard 52-card deck, with 28 (54%) cards dealt in 7 build stacks and the other 24 (46%) cards placed in a pile, as can be seen in Figure 1. The goal of the game is to order all cards from the ace to the king on the 4 corresponding suit stacks. Cards can be moved on top of other build stacks if their rank is one lower than the current top card and of the opposite color. Cards can be requested from the pile to be placed on the talon (Figure 1).

### Study Objective

Given the popularity of Klondike Solitaire among the older population, and given the need for engaging, ecologically valid, scalable tools to assist in the screening and monitoring of MCI, this paper aims to investigate the feasibility of Klondike Solitaire game sessions to yield digital biomarkers of MCI. To this end, the study comprised the following investigations:

1. an exploration of the digital biomarkers of cognitive performance, based on the player actions (PAs) of Klondike Solitaire and
2. an evaluation of the candidate digital biomarkers captured in Klondike Solitaire to measure the differences between healthy older adults and older adults living with MCI.

## Methods

### Crafting Candidate Digital Biomarkers in Klondike Solitaire

To explore the potential digital biomarkers of cognitive performance in Klondike Solitaire, we first conducted an expert consensus study, involving 11 experts. In this first part of the paper, we discuss the 3 steps taken to compile a final list of 23 candidate digital biomarkers.

### Step 1: Defining PAs

To transform gameplay into PAs, a methodical approach was applied. In all, 4 researchers in the field of human-computer interaction carried out the following tasks. First, the literature on Klondike Solitaire was studied, ranging from scientific work [72-78] to more general sources [79-81]. Afterward, the game was played for a minimum of 10 sessions of 30 minutes by each of the researchers. Combining the theoretical background with this practical experience, the experts independently drafted a list of game events, which was then reviewed as a team. This list was iterated 3 times until no more game events were found. The game events included, but were not limited to, game outcomes (eg, the game was won or lost), correct player moves (eg, the player moves a card among the build stacks), and erroneous player moves (eg, the player places cards on other cards such that they are not in descending order on the build stack).

These game events were then converted to PAs; they were described as an action of the player rather than as an event of the game. Next, all these PAs were transformed into their negative equivalents, for example, *The player takes little time to think of a move* was reworded as *The player takes a lot of time to think of a move*. The reason for this was 2-fold. It enabled inverse PAs (the positive and negative equivalents, eg, moving cards fast or moving them slowly) to be combined, reducing the rating complexity for the professionals. Furthermore, the negative equivalent aimed to facilitate the rating process because impaired cognition leads to reduced performance in gameplay. After this step was completed, 21 PAs (Table 1) were defined for evaluation.

**Table 1:** Average of the experts’ ratings for each player action and cognitive function.

Player actions	Mental flexibility, mean (SD)	Inhibitory control, mean (SD)	Working memory, mean (SD)	Selective attention, mean (SD)	Visuospatial ability, mean (SD)	Object recognition, mean (SD)	Apraxia, mean (SD)	Cognitive planning, mean (SD)	Processing speed, mean (SD)
PA^a^ 1. Player takes a lot of time to think of a move.	1.64 (1.12)	0.73 (1.01)	1.82 (0.4)	1.55 (0.82)	1.18 (0.98)	1.27 (0.79)	0.27 (0.47)	*2.45*^b^ (0.52)	*2.55* (0.82)
PA 2. Player takes a lot of time to move the card.	0.73 (1.01)	0.73 (1.1)	0.64 (0.5)	0.64 (0.67)	1.45 (1.04)	0.64 (0.67)	1.64 (0.92)	0.91 (0.83)	*2.09* (1.04)
PA 3. Player does not move a suitable card from the talon to the suit stack.	*2.27* (0.79)	0.73 (0.9)	*2* (0.77)	*2.55* (0.69)	1.45 (0.93)	1.64 (0.67)	0.73 (1.01)	*2.18* (0.75)	0.91 (1.04)
PA 4. Player does not move a suitable card from the build stack to the suit stack.	1.82 (0.98)	0.91 (0.83)	1.82 (0.75)	*2.36* (0.67)	1.36 (0.92)	1.36 (0.92)	0.27 (0.47)	1.73 (0.79)	1 (1)
PA 5. Player does not move a suitable card from the talon to the build stack.	*2.18* (0.75)	1.27 (0.9)	1.91 (0.7)	*2.64* (0.67)	1.55 (1.04)	1.73 (0.79)	0.18 (0.4)	*2.09* (0.94)	0.82 (1.08)
PA 6. Player does not move a suitable card from 1 build stack to another build stack.	*2.36* (0.81)	1.09 (0.94)	*2* (0.77)	*2.45* (0.69)	1.45 (0.93)	1.64 (0.92)	0.18 (0.4)	*2.18* (0.98)	0.73 (1.01)
PA 7. Player does not place an ace immediately on an empty suit stack.	1.27 (1.01)	0.73 (1.01)	*2.18* (0.4)	*2.36* (0.92)	1 (0.77)	1.18 (1.17)	0.45 (0.69)	*2.09* (0.7)	1.09 (1.04)
PA 8. Player does not place a king on an empty build stack.	1.45 (1.13)	0.73 (0.9)	*2* (0.77)	*2.27* (0.9)	1 (0.77)	1.55 (1.13)	0.36 (0.67)	*2.09* (0.7)	1 (0.89)
PA 9. Player moves cards without benefit (eg, moving a jack from 1 queen to another).	1.45 (1.04)	1.82 (0.98)	*2.18* (1.17)	1.64 (1.03)	0.82 (0.75)	1.45 (1.21)	0.18 (0.4)	*2.27* (1.01)	0.45 (0.52)
PA 10. Player flips a lot through the pile.	*2* (0.89)	*2.55* (0.69)	1.73 (1.01)	1.82 (1.08)	1 (0.89)	1.45 (1.13)	1 (1)	*2.09* (1.04)	0.91 (0.7)
PA 11. Player moves a card onto a card with the same color.	1.73 (1.1)	*2.55* (0.52)	*2.18* (0.98)	*2.18* (0.98)	1 (1)	1.82 (1.17)	0.27 (0.65)	1.36 (0.81)	0.45 (0.69)
PA 12. Player moves a card to another card with the wrong number (eg, placing a 5 on a 10).	1.18 (1.08)	*2* (1)	*2.27* (0.9)	1.91 (0.94)	1.09 (1.04)	2.09 (0.94)	0.45 (0.69)	1.45 (0.93)	0.36 (0.5)
PA 13. Player selects the cards with a very bad precision (taps on edge of card or next to the card).	0.45 (0.69)	0.73 (0.79)	0.27 (0.47)	0.64 (0.81)	*2.27* (0.9)	0.82 (0.75)	*2.27* (0.79)	0.45 (0.82)	0.45 (0.69)
PA 14. Player starts tapping on the playfield with no apparent target (with short intervals, fidget tapping).	0.73 (0.79)	*2.27* (1.01)	0.27 (0.47)	0.82 (0.87)	0.73 (0.9)	0.45 (0.52)	1.55 (1.29)	0.91 (1.04)	0.73 (0.9)
PA 15. Player presses the undo button a lot.	1.82 (0.6)	*2.45* (0.69)	1.73 (1.1)	1.36 (1.12)	0.64 (0.67)	0.64 (0.67)	0.73 (1.01)	*2.27* (1.01)	1.27 (1.1)
PA 16. Player requests a lot of hints.	1.91 (1.04)	1.73 (1.01)	*2* (1)	1.45 (0.93)	0.64 (0.81)	1 (0.77)	0.45 (0.69)	*2.27* (1.01)	1.18 (0.75)
PA 17. Player takes a very long time to finish games.	*2.18* (1.25)	1 (1.34)	*2.18* (0.75)	1.64 (1.21)	1.09 (0.83)	1.18 (0.98)	0.91 (0.83)	*2.64* (0.5)	*2.91* (0.3)
PA 18. Player does not have a high score in the game.	*2.18* (0.98)	*2* (1)	*2.36* (1.03)	1.91 (1.04)	1.45 (0.93)	1.36 (0.92)	0.91 (0.94)	*2.27* (0.9)	1.55 (1.04)
PA 19. Player does not win a lot of games (low win ratio).	*2.36* (0.67)	1.82 (1.08)	*2.64* (0.5)	2 (1)	1.36 (0.92)	1.18 (0.87)	1 (0.89)	*2.82* (0.4)	1.64 (0.81)
PA 20. Player’s scores in different games vary greatly.	*2.27* (1.1)	1.64 (1.12)	*2.27* (0.79)	*2.36* (1.12)	0.73 (0.9)	0.73 (0.9)	0.64 (0.92)	*2.18* (1.08)	1.82 (1.08)
PA 21. Player’s win ratio decreases rapidly as the game’s level of difficulty increases.	*2.36* (0.67)	1.91 (0.94)	*2.64* (0.67)	*2.18* (0.87)	1.18 (0.87)	1.09 (0.94)	0.82 (0.87)	*2.64* (0.81)	1.64 (1.03)

^a^PA: player action.

^b^Values scoring moderately strong are in italics.

### Step 2: Defining Cognitive Functions

**Figure 2:**
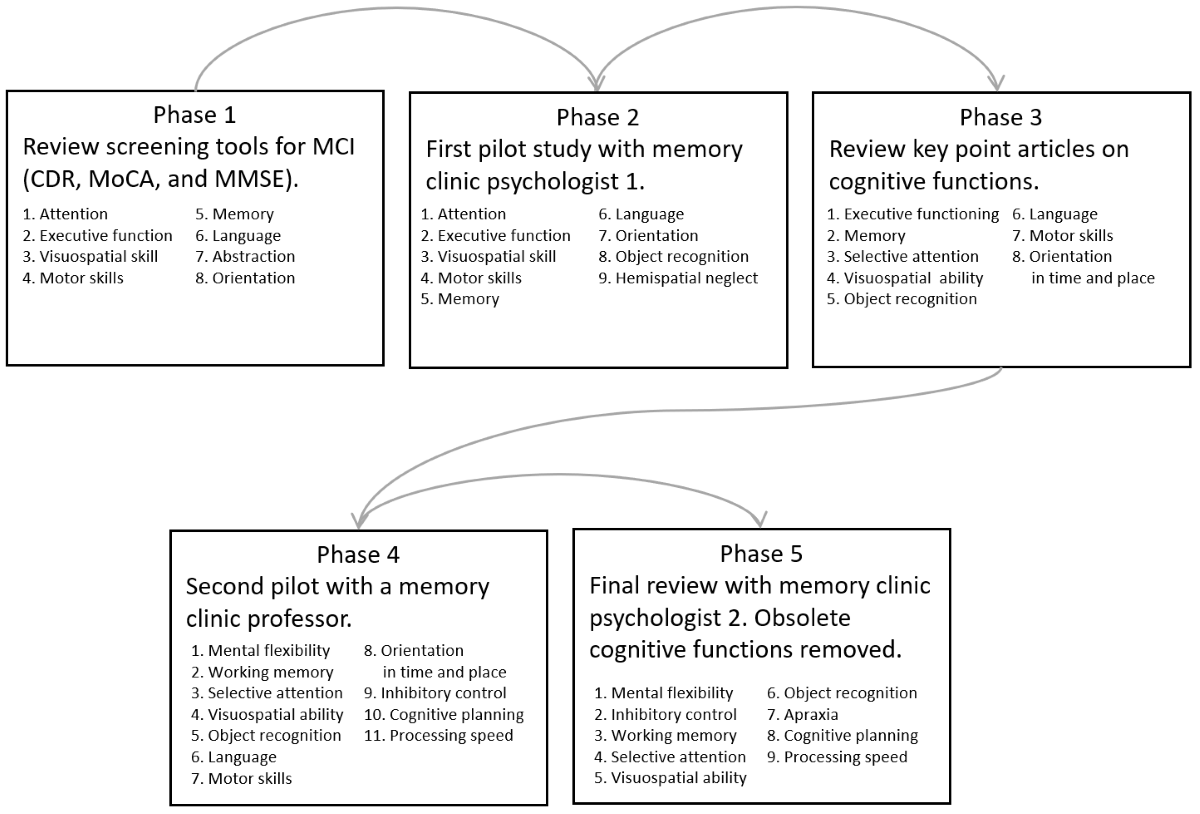
The 5 phases through which the cognitive functions were defined. CDR: Clinical Dementia Rating, MoCA: Montreal Cognitive Assessment, MMSE: Mini-Mental State Examination.

A set of cognitive functions was drafted in 5 phases (Figure 2). A first draft was prepared (phase 1), beginning with the cognitive functions measured using the screening tests used most often for MCI [36,37,82]. In phase 2, during a trial with a psychologist, we replaced abstraction with object recognition to more clearly indicate the problems with finding suitable cards, based on key articles on cognitive aging and cognition [83-87]. Next (phase 3), to better delineate attention, it was specified as selective attention. In phase 4, a pilot study was conducted with an expert on memory and age-related disorders (with 23 years of clinical and research experience). On the basis of this pilot testing, it was decided to split executive functioning into inhibitory control, cognitive planning, and mental flexibility. Memory was further specified as working memory and lack of motor skills as apraxia. In the final iteration, cognitive functions ostensibly not present in Klondike Solitaire, that is, orientation in time and space, as well as language, were removed to reduce the rating complexity. This resulted in a set of 9 cognitive functions (Figure 2, phase 5).

### Protocol for Rating Functions and Actions

#### Overview

In the next step, the experts were asked to rate the extent to which each PA was related to a specific cognitive function.

These experts were recruited from 2 leading memory clinics in Belgium using a snowball sampling method. Of the 11 experts, 3 (27%) held Doctor of Medicine degrees and were experienced in treating cognitive decline, whereas the other 8 (73%) were neuropsychologists; 7 (64%) were women; and 4 (36%) were men. The average age of the experts was 45 (SD 13.3) years, and their average working experience was 20 (SD 14) years. In all, 3 coauthors of this paper (LVA, PD, and FFB) also participated as experts. None of the experts were compensated for participating in the study.

Before they began the rating process, the experts received a standardized introduction comprising a video that explained all concepts of the game [88], a video that visualized all 21 PAs [89], and a document that provided explanatory notes for all 9 cognitive functions. The aim of providing this introduction was to prevent confusion regarding game terminology, interpretation of PAs, and cognitive functions. The introduction also included a delineation of the target group to persons living with multiple-domain aMCI. The experts could revisit the videos and document at any time.

Next, each expert received a coding sheet in which they could map the 21 PAs to the 9 cognitive functions. Each cell had to be filled in according to the following 4-point scale:

0: This cognitive function has no significant correlation to the PA. 1: This cognitive function correlates weakly to the PA. 2: This cognitive function correlates moderately to the PA. 3: This cognitive function correlates strongly to the PA.

Finally, they were also given the choice to explain their train of thought in an optional clarification column.

#### Expert Agreement on PAs and Cognitive Functions

The intraclass correlation (ICC) for each PA as variables of interest with cognitive functions was computed. In addition, we computed ICCs for each of the cognitive functions as variables of interest, with all PAs considered as observations [90]. All calculations were executed using SPSS software (version 23.0; IBM Corp) [91]. The ICC was calculated to verify the rater agreement [92] on PAs and cognitive functions based on a 2-way, random, fully crossed design with type consistency [93]. According to the criteria described in the study by Koo et al [94], ICCs below 0.5 are indicative of low reliability, ICCs between 0.5 and 0.75 are indicative of moderate reliability, ICCs between 0.75 and 0.9 are indicative of good reliability, and ICCs above 0.9 are indicative of excellent reliability.

We found that the ICCs for all PAs scored higher than 0.75, suggesting good to excellent reliability according to the study by Koo et al [94]. With the exception of 4 cognitive functions (ie, mental flexibility, visuospatial ability, object recognition, and apraxia with scores of 0.68, 0.42, 0.66, and 0.71, respectively), the ICCs of the cognitive functions scored higher than 0.75, suggesting good to excellent reliability.

#### Cognitive Functions Present in Klondike Solitaire

An overview of the associations between individual PAs and cognitive functions, according to the expert mapping, is presented in Table 1. In addition, an overview of all ICCs with 95% CIs is presented in Multimedia Appendix 1. Each PA was related by the experts to one or more cognitive functions with an average association above two, which indicated a moderate to strong relationship with the cognitive functions. Similarly, each cognitive function was associated with at least one PA, with an average association of more than two.

### Step 3: Defining Candidate Digital Biomarkers

These PAs were captured through the game as potential digital biomarkers, that is, measurable factors of the game, such as score, duration of the game, and detailed moves. These candidate digital biomarkers were enriched with additional information about the game. This contextualization is important to ensure an unambiguous interpretation of the cognitive information derived from the gameplay. For example, whereas a game played with many moves on the pile may indicate that a player progressed in the game, it may indicate equally that the player did not realize that they were stuck. By calculating the percentage of pile moves by dividing the number of pile moves by the total number of moves, a more informative metric can be obtained. In this manner, 23 potential digital biomarkers were defined that we further classified into 1 of 6 categories: time-based, performance-based, error-based, execution-based, auxiliary-based, and result-based. Time-based digital biomarkers are biomarkers related to the speed of PAs. Performance-based digital biomarkers are biomarkers related to optimal gameplay (ie, if the game was played according to strategies that ensure optimal performance). Error-based digital biomarkers relate to making incorrect moves according to the rules of Klondike Solitaire. Auxiliary-based digital biomarkers are interactions that are not part of the core gameplay (ie, requesting undo moves and hints). Execution-based digital biomarkers relate to the accuracy in moving cards and the presence of accidental taps. Finally, result-based digital biomarkers are biomarkers that evaluate the final outcome of the game (eg, how far did the participant get in the game). An overview of all digital biomarkers and their contextualizations is presented in Table 2.

**Table 2:** Digital biomarkers related to the player actions in Klondike Solitaire^a^.

Related PA^b^		Digital biomarker		Description		Contextualization		Value	
**Time-based**									
	PA 1	Think time		Time spent thinking of a move. Defined as the time necessary to find and touch a suitable card		Average (SD)		Number (ms)	
	PA 2	Move time		Time spent moving cards. Defined as the time necessary to move a suitable card to the destination		Average (SD)		Number (ms)	
	PA 1, PA 2	Total time		Total time to make a move. Defined as the combination of think time and move time		Average (SD)		Number (ms)	
**Performance-based**									
	PA 3	Final β error		Whether there were still moves possible when quitting a game		None		Boolean	
	PA 3, PA 4, PA 5, PA 6	β error		Number of pile moves made with moves remaining on the board divided by the total number of pile moves		Percentage		0%-100%	
	PA 7	Ace β error		Number of missed opportunities to place an ace on the suit stacks divided by the total number of game moves		Percentage		0%-100%	
	PA 8	King β error		Number of missed opportunities to place a king on an empty spot divided by the total number of game moves		Percentage		0%-100%	
	PA 10	Pile move		Number of pile moves divided by the total number of board moves		Percentage		0%-100%	
**Error-based**									
	PA 11, PA 12		Successful move		Number of successful moves divided by the total number of board moves		Percentage		0%-100%
	PA 11, PA 12		Erroneous move		Number of erroneous moves divided by the total number of board moves		Percentage		0%-100%
**Execution-based**									
	PA 13	Accuracy		Accurateness of selecting a card, defined by how close to the center a card was touched		Average (SD)		0%-100%	
	PA 14	Taps		Number of actuations on nonuser interface elements		None		Number	
**Auxiliary-based**									
	PA 15	Undo move		Number of undo moves requested.		Percentage		0%-100%	
	PA 16	Hint move		Number of hints requested		Percentage		0%-100%	
**Result-based**									
	PA 17	Game time		Total time spent playing a game		None		Number (ms)	
	PA 18	Score		Final score of a game		None		Number	
	PA 19	Solved		Whether the game was completed. Indicator of how successfully the game was played		None		Boolean	
	PA 19	Cards moved		Number of cards selected for each move. An additional indicator of how successfully the game was played; as games of Klondike Solitaire progress, longer stacks of cards are moved		Average (SD)		Number	

^a^Player actions (PAs) 20 and 21 were not captured because the single-point-in-time setup would not allow a comparison of scores and win ratios with ranging difficulty over time. In addition, PA 9 was not tested because the current software would not allow for detection of these moves.

^b^PA: player action.

### Evaluating Digital Biomarkers

The aim of this second study is to explore the potential of these candidate digital biomarkers of cognitive performance. Relying on 46 participants, we captured data and performed a GLMM analysis to examine the differences between healthy participants and those diagnosed with MCI.

### Participants

In total, 46 participants—23 (50%) healthy participants and 23 (50%) with MCI—were enrolled. The older adults with MCI were recruited by 2 leading memory clinics in Belgium. Healthy participants were recruited from multiple senior citizen organizations, using a snowball sampling method. All healthy participants were aged 65 years or older, were fluent in written and verbal Dutch, had 20/20 (corrected) vision, no motor impairments, and lived independently or semi-independently at home, in a service flat, or at a care home. The exclusion criteria for healthy participants were subjective-memory concerns expressed by the participant, caretaker, or clinician. In addition, they were screened using the MMSE, MoCA, and CDR Scale. To minimize the risk of including potential individuals with MCI among the healthy participants, cutoff scores of 27 on the MMSE, 26 on the MoCA, and 0 on the CDR Scale were enforced. The participants living with MCI had been formally diagnosed with multiple-domain aMCI by 1 of the 2 collaborating memory clinics, based on the diagnostic criteria described in the study by Petersen [95]. Participants with MCI were excluded if they scored lower than 23 on the MMSE to avoid including participants who could be on the borderline between the diagnosis of MCI and dementia. All recruited participants had prior experience with Klondike Solitaire. This familiarity with the rules was imperative because participants with MCI may have problems with memorizing and recalling new game rules because of short-term memory issues. Moreover, the rationale underlying this study is that it is imperative to draw from games already played and enjoyed by participants and where the rules are crystallized in their memory. Demographic and basic neuropsychological data of both groups are presented in Table 3.

**Table 3:** Demographic and neuropsychological data (N=46).

			Healthy participants (n=23)		Participants diagnosed with MCI^a^ (n=23)
Age (years), mean (SD)			70 (5.4)		80 (5.2)
**Education (ISCED^b^ level) [96], n (%)**					
	Levels 1-2	5 (22)		4 (17)	
	Levels 3-4	7 (30)		13 (57)	
	Levels 5-6	11 (48)		6 (26)	
**Sex, n (%)**					
	Female	11 (47)		13 (57)	
	Male	12 (53)		10 (43)	
**Tablet proficiency, n (%)**					
	Daily	12 (52)		3 (13)	
	Weekly	2 (9)		2 (9)	
	Monthly	0 (0)		2 (9)	
	Yearly or less	2 (9)		1 (4)	
	Never	7 (30)		15 (65)	
**Klondike Solitaire proficiency, n (%)**					
	Daily	3 (13)		7 (30)	
	Weekly	6 (26)		8 (35)	
	Monthly	3 (13)		2 (9)	
	Yearly or less	11 (47)		6 (26)	
	Never	0 (0)		0 (0)	
MMSE^c^ score, mean (SD)			29.61 (0.65)		26.17 (1.75)
MoCA^d^ score, mean (SD)			28.09 (1.28)		N/A^e^
CDR^f^ Scale score, mean (SD)			0 (0)		N/A

^a^MCI: mild cognitive impairment.

^b^ISCED: International Standard Classification of Education.

^c^MMSE: Mini-Mental State Examination.

^d^MoCA: Montreal Cognitive Assessment.

^e^N/A: not applicable.

^f^CDR: Clinical Dementia Rating.

### Data Collection Tools

All game sessions were completed on a Lenovo Tab3 10 Business tablet (Lenovo Group Ltd) running Android 6.0 (Google LLC). A Solitaire app created by Bielefeld [97] under the Lesser General Public License 3 was modified to capture and store game metrics that served as building blocks for the digital biomarkers of cognitive performance.

### Data Collection Procedure

Each observation was made between 9 AM and 5 PM in the home environment of the participant to ensure a familiar and comfortable environment. Each observation took between 2 and 3 hours and consisted of 2 main parts:

1. a game session where game-based digital biomarkers of Klondike Solitaire were collected on a tablet and
2. a neuropsychological examination where cognitive information was collected.

Each game session started with a standardized 5-minute introduction during which the tablet, the game mechanics, and possible touch interactions were explained. This was followed by a practice game, identical for all participants, where questions to the researcher were allowed and the participant could get used to the touch controls. Data from this practice game were not used for analysis. After this practice game, each participant played 3 games in succession. The order and games were identical across all participants. All games were purposefully chosen through prior playtesting, in that they were solvable and varied in difficulty level. During these 3 games, no questions were allowed, and gameplay continued until the participants either finished the game or indicated that they deemed further progress impossible. All game sessions and cognitive evaluations were conducted by the same researcher to avoid differences arising from researcher bias.

### Ethical Statement

This study is in accordance with the declaration of Helsinki and General Data Protection Regulation compliant. Ethical approval was provided by the ethics committee of UZ/KU Leuven, Belgium (CTC S59650). Because of the fragile nature of our participants’ health, utmost care was taken when providing information to them about the game sessions. The tests were conducted only after we received written informed consent.

### Statistical Analysis

To assess the difference between the healthy participants and those diagnosed with MCI, a GLMM analysis was performed using R software (The R Foundation for Statistical Computing) [98] with the lme4 library [99]. Concerning the design of our GLMM, the fixed effects consisted of MCI, age, tablet proficiency, and Klondike Solitaire proficiency. The random effects were modeled as random intercepts for game seed and participant. In addition, by-participant random slopes for the effect of MCI were modeled.

Continuous digital biomarkers (eg, think time average) were modeled using a GLMM with the identity link function. Binary outcomes (eg, solved or not solved) were modeled using a GLMM with the logit link function. The significance of the effect of MCI was determined using the likelihood ratio test, which compares the model with a model without the effect of MCI, both estimated without restricted maximum likelihood [100,101]. Assumptions of homoscedasticity and normality were visually inspected using residual plots. To provide supplemental information on the fit of the models, the marginal *R*² and the conditional *R*² were given as specified in the study by Nakagawa and Schielzeth [102]. Given the exploratory nature of this study, we did not correct for family-wise inflation error [103].

## Results

### Overview

**Figure 3:**
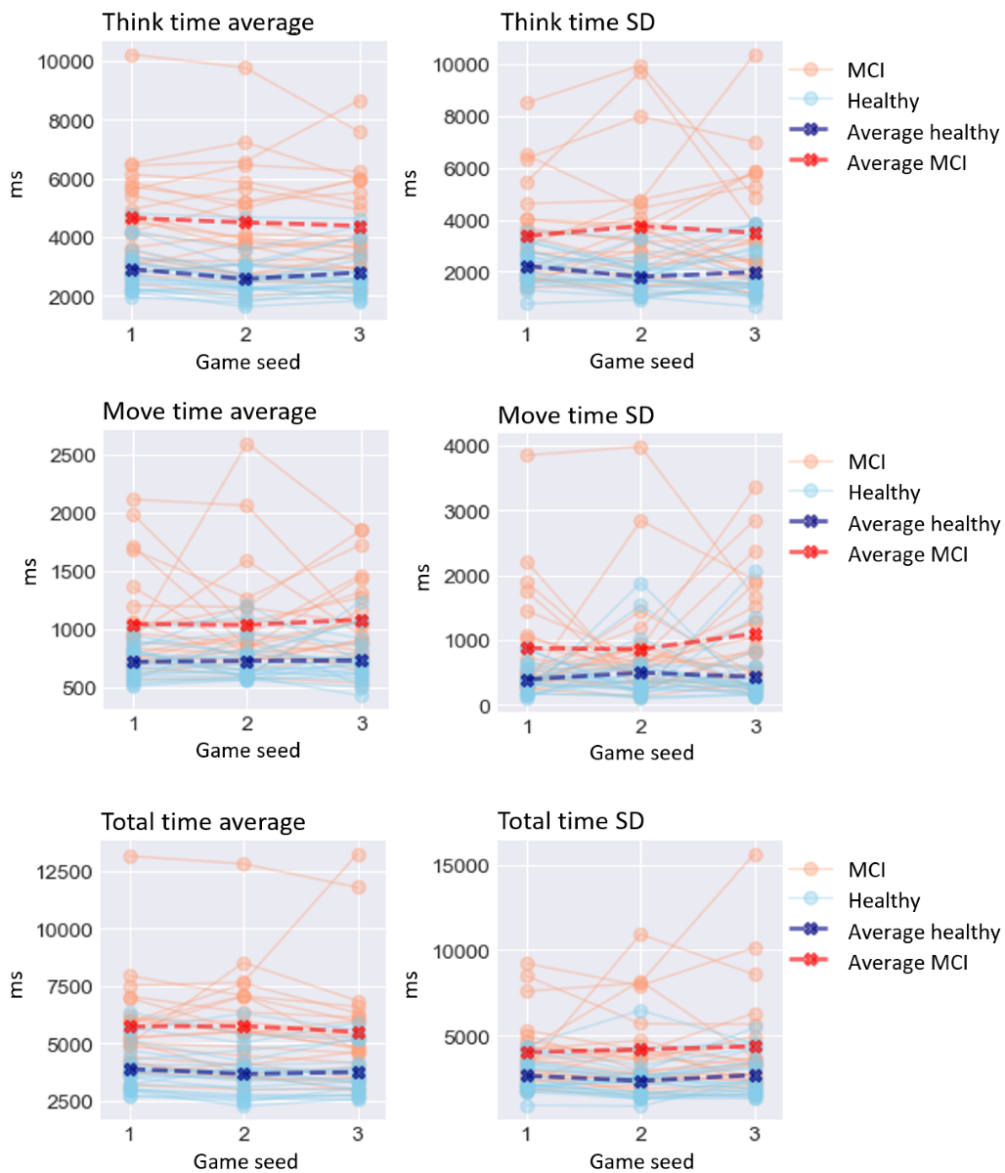
Performance on time-based digital biomarkers for both groups. MCI: mild cognitive impairment.

**Figure 4:**
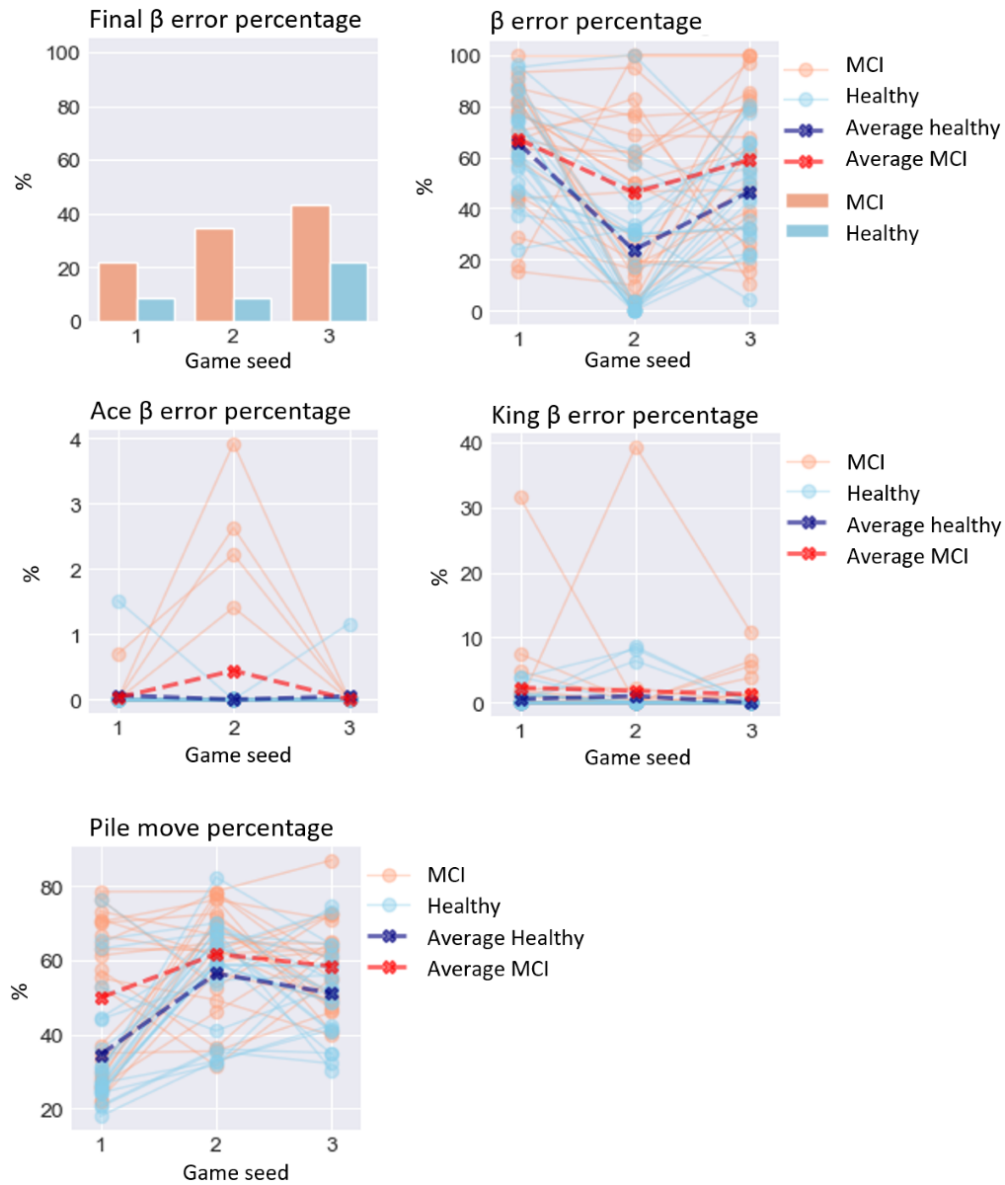
Performance on performance-based digital biomarkers for both groups. MCI: mild cognitive impairment.

**Figure 5:**
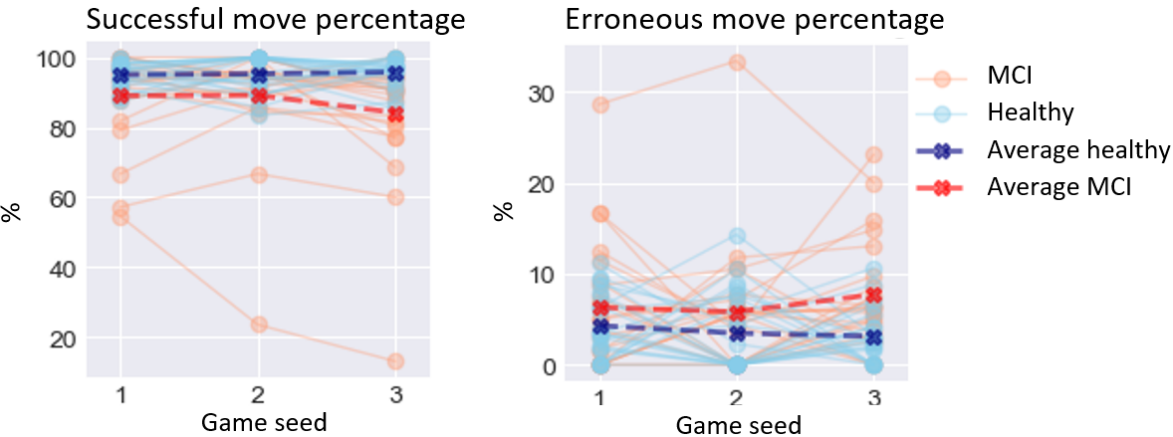
Performance on error-based digital biomarkers for both groups. MCI: mild cognitive impairment.

**Figure 6:**
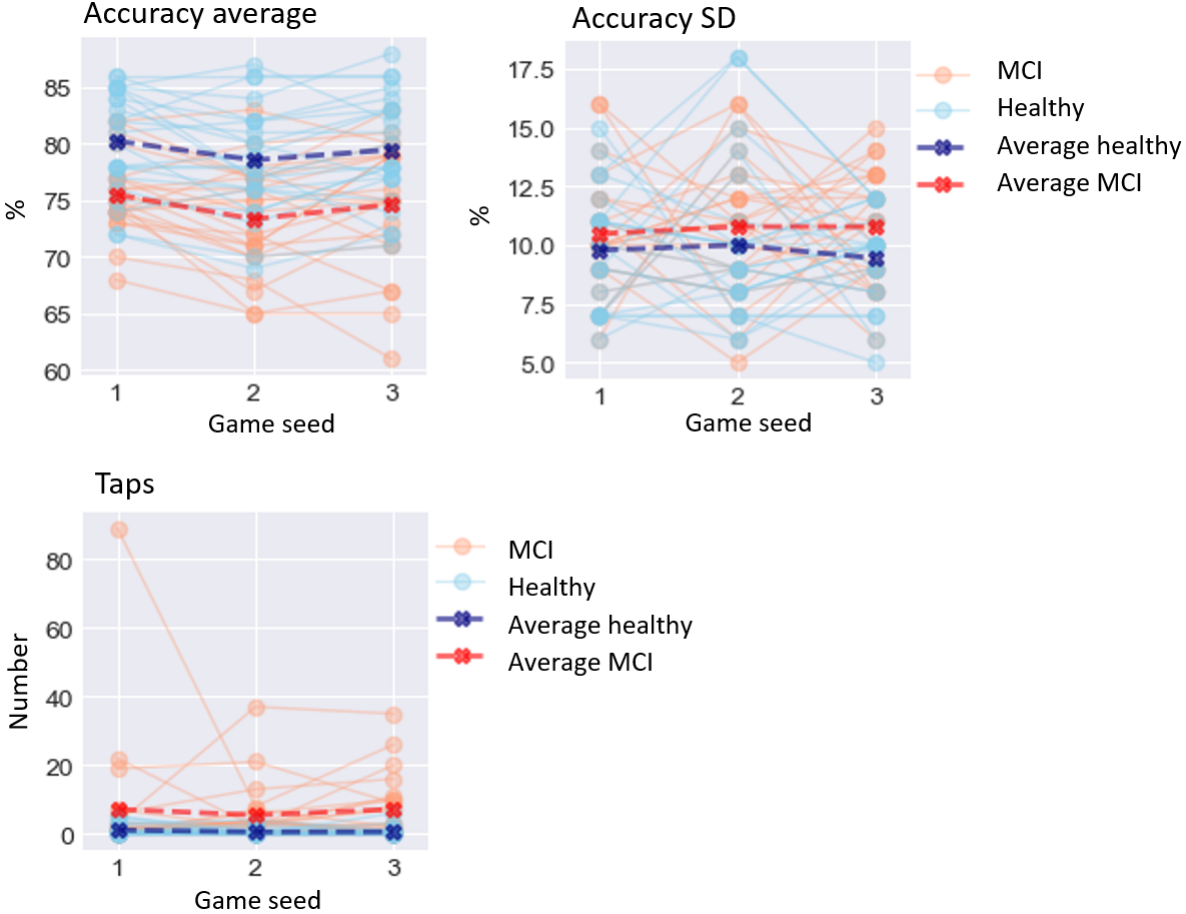
Performance on execution-based digital biomarkers for both groups. MCI: mild cognitive impairment.

**Figure 7:**
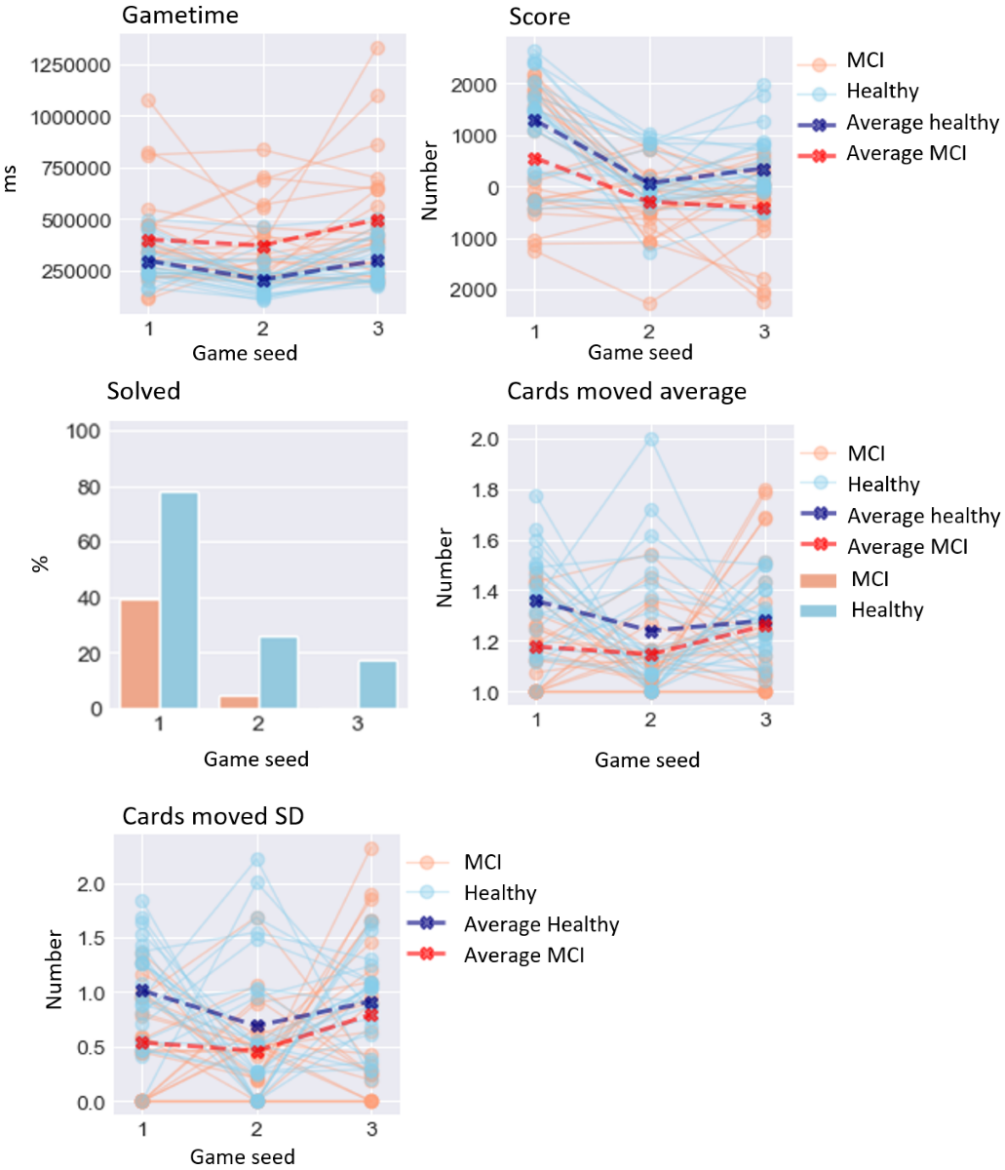
Performance on result-based digital biomarkers for both groups. MCI: mild cognitive impairment.

**Figure 8:**
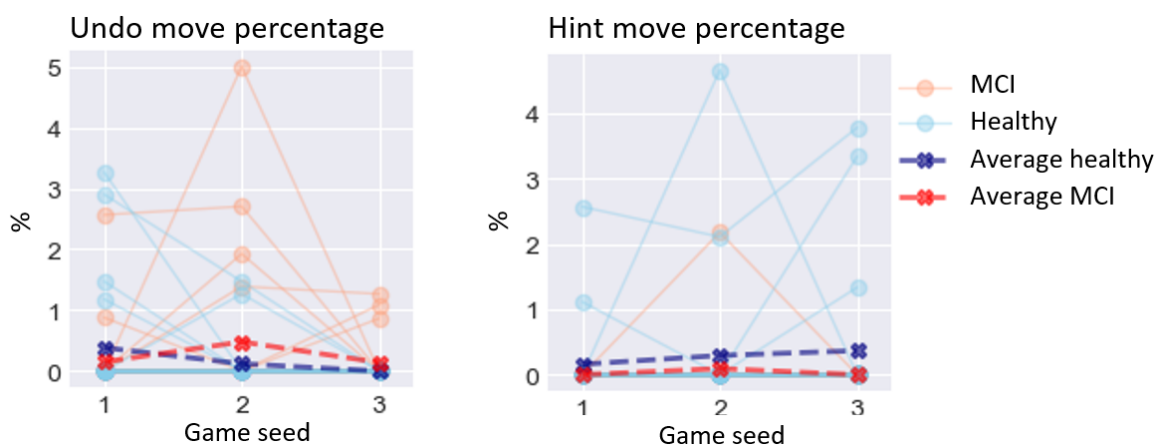
Performance on auxiliary-based digital biomarkers for both groups. MCI: mild cognitive impairment.

The results of the GLMM analysis on the effect of MCI are presented below. A visualization of digital biomarker performance for all groups across all games is presented in Figures 3-8. A summary is presented in Table 4.

**Table 4:** Generalized linear mixed model analysis results for each digital biomarker.

Digital biomarker			Value, constant (SD)		Value, β (SD)		*P* value (χ^2^)		Value, *R*²m (*R*²c)
**Time-based**									
	Think time average	–1371.778 (1415.444)		1119.947 (405.815)		.006		0.416 (0.904)	
	Think time SD	–814.527 (1720.073)		1112.533 (490.53)		.02		0.211 (0.655)	
	Move time average	–508.575 (373.89)		156 (95.547)		.1		0.257 (0.579)	
	Move time SD	–856.605 (847.852)		323.599 (202.032)		.1		0.137 (0.419)	
	Total time average	–912.419 (2149.177)		1278.263 (573.839)		.02		0.318 (0.870)	
	Total time SD	206.569 (2676.062)		1315.598 (673.665)		.04		0.176 (0.715)	
**Performance-based**									
	Final β error	–7.233 (4.131)		0.435 (0.922)		.65		0.096 (0.068)	
	β error percentage	–7.203 (33.849)		6.108 (6.879)		.36		0.089 (0.371)	
	Ace β error percentage	–0.132 (0.629)		0.051 (0.137)		.73		0.023 (0.209)	
	King β error percentage	–3.682 (5.918)		0.907 (1.323)		.48		0.028 (0.230)	
	Pile move percentage	71.759 (24.052)		13.333 (4.88)		.006		0.097 (0.513)	
**Error-based**									
	Successful move percentage	87.486 (9.443)		–8.913 (3.595)		.02		0.104 (0.795)	
	Erroneous move percentage	9.486 (6.529)		3.624 (1.651)		.03		0.081 (0.466)	
**Execution-based**									
	Accuracy average	92.134 (9.167)		–3.817 (1.903)		.04		0.246 (0.805)	
	Accuracy SD	4.519 (3.746)		0.137 (0.772)		.85		0.056 (0.196)	
	Taps	–5.113 (10.704)		5.334 (2.762)		.05		0.098 (0.500)	
**Auxiliary-based**									
	Undo move percentage	0.228 (0.955)		0.135 (0.205)		.49		0.008 (0.151)	
	Hint move percentage	–0.58 (0.87)		–0.311 (0.204)		.12		0.046 (0.491)	
**Result-based**									
	Gametime	–167427.7 (187325.5)		93211.27 (53699.25)		.08		0.198 (0.690)	
	Score	29.03 (1389.752)		–744.433 (286.576)		.009		0.105 (0.612)	
	Solved	–2.954 (4.578)		–2.63 (1.007)		.008		0.186 (0.152)	
	Cards moved average	1.111 (0.262)		–0.119 (0.054)		.03		0.061 (0.093)	
	Cards moved SD	0.135 (0.705)		–0.38 (0.147)		.009		0.072 (0.152)	

### Time-Based Digital Biomarkers

For time-based digital biomarkers (Figure 3), MCI significantly affected think time average (*χ*^2^_1_=7.7; *P*=.006), increasing it by 1119.947 ms (SD 405.81). Equally significantly, MCI affected think time SD (*χ*^2^_1_=5.1; *P*=.02), increasing it by 1112.533 ms (SD 490.53). However, MCI did not significantly affect move time average (*χ*^2^_1_=2.7; *P*=.10) or move time SD (*χ*^2^_1_=2.6; *P*=.10). MCI significantly affected total time average (*χ*^2^_1_=5.2; *P*=.02), increasing it by 1278.263 ms (SD 573.84), and total time SD (*χ*^2^_1_=4.1; *P*=.04), increasing it by 1315.598 ms (SD 673.67).

### Performance-Based Digital Biomarkers

For performance-based digital biomarkers (Figure 4), MCI did not significantly affect final β error percentage (*χ*^2^_1_=0.2; *P*=.65). Equally, MCI did not significantly affect β error percentage (*χ*^2^_1_=0.8; *P*=.36), ace β error percentage (*χ*^2^_1_=0.1; *P*=.73), or king β error percentage (*χ*^2^_1_=0.5; *P*=.48). MCI significantly affected pile move percentage (*χ*^2^_1_=7.5; *P*=.006), increasing it by 13.333% (4.88).

### Error-Based Digital Biomarkers

For error-based digital biomarkers (Figure 5), MCI significantly affected successful move percentage, (*χ*^2^_1_=5.9; *P*=.02), lowering it by 8.913% (SD 3.6). MCI also significantly affected erroneous move percentage, (*χ*^2^_1_=4.8; *P*=.03), increasing it by 3.624% (SD 1.65).

### Execution-Based Digital Biomarkers

For execution-based digital biomarkers (Figure 6), MCI significantly affected accuracy average (*χ*^2^_1_=4.1; *P*=.04), lowering it by 3.817% (SD 1.9). MCI did not significantly affect accuracy SD (*χ*^2^_1_=0.04; *P*=.85) or taps (*χ*^2^_1_=3.8; *P*=.05).

### Result-Based Digital Biomarkers

For result-based digital biomarkers (Figure 7), MCI did not significantly affect gametime (*χ*^2^_1_=3.1; *P*=.08). MCI significantly affected solved (*χ*^2^_1_=6.9; *P*=.008), lowering it by 2.63 (SD 1.01). MCI also significantly affected cards moved average (*χ*^2^_1_=4.9; *P*=.03), lowering it by 0.119 cards (SD 0.05), and cards moved SD (*χ*^2^_1_=6.7; *P*=.009), lowering it by 0.38 cards (SD 0.15).

### Auxiliary-Based Digital Biomarkers

With regard to auxiliary-based digital biomarkers (Figure 8), none of these candidate biomarkers reached significance: undo move percentage (*χ*^2^_1_=0.4; *P*=.49) and hint move percentage (*χ*^2^_1_=2.4; *P*=.12).

## Discussion

### Overview

MCI is a neurological disorder that is linked to an increased risk of developing dementia. As such, early detection of cognitive deterioration is essential for timely diagnosis and for allowing tailored care and treatment. Collecting digital biomarkers through COTS games may help by providing cognitive information through behavior traces of activities already integrated into the daily life of older adults. In this study, we investigated in particular whether Klondike Solitaire game sessions could yield digital biomarkers. In the paragraphs below, we discuss our findings and reflect on the different potential digital biomarkers, their relationship with cognitive functions, and the ethical implications of their use for cognitive assessment purposes.

### Dissecting Digital Biomarkers

Of the 23 candidate digital biomarkers, 12 (52%) differed significantly between older adults with MCI and a healthy control group. This supports the use of digital card games for monitoring cognitive performance and possibly detecting differences in cognitive performance caused by MCI.

Although the overall findings are promising, not all candidate biomarkers performed equally. In the case of time-based digital biomarkers, the biomarkers related to coming up with a move—think time average and think time SD—were significantly affected by MCI. In contrast, the biomarkers related to the actual physical movement of cards—move time average and move time SD—were not significantly affected. Total time average (*P*=.02), which consists of move time as well as think time, was significantly affected; yet, it was less significant than think time average (*P*=.006). These results indicate that segmenting in-game actions can be beneficial because they can more accurately isolate cognitive functions such as praxis and cognitive planning.

In the case of performance-based digital biomarkers, in contrast with expectations, none of the biomarkers related to β errors were proven to differ significantly. Upon rewatching gameplay, it became clear that there were two different types of β errors: strategic and unintentional. However, because of the current configuration of the app, it was impossible to discriminate between the two types. This is discussed further in the *Limitations* section. In contrast, pile move percentage was proven to differ significantly. This may indicate that older adults with MCI may not recognize the same cards being returned as quickly as their healthy counterparts.

Equally, the results indicated that participants with MCI made more mistakes because both error-based digital biomarkers (ie, successful move percentage and erroneous move percentage) were significant. In contrast, none of the auxiliary-based digital biomarkers differed significantly. Upon inspecting the data, it was noted that neither group consistently used these functionalities, which may have contributed to the lack of significance.

Finally, of the 5 digital biomarkers in the result-based category, 4 (80%) were significant, 3 (60%) with *P*<.01 (ie, score, solved, and cards moved SD). The outcome of these measures is the result of a series of consequent moves, each of them being potentially crucial to complete the game. For example, a lapse in attention or executive functioning can cause important moves to be overlooked, in turn making the game unsolvable. Although overall gametime was not significant, this can be explained by the fact that time spent in the game by itself does not indicate a lesser performance. Time-based digital biomarkers, which are equally measures of time but contextualized with the number of moves made, show more significant results (ie, think time average, think time SD, total time average, and total time SD), stressing the importance of contextualization.

In sum, our findings are in accordance with those of the study by Jimison et al [8], which used FreeCell, a Solitaire variant, to compare cognitive performance between a group of people living with MCI and a healthy control group. Using card gameplay, we can discern older adults with MCI from a healthy control group. Moreover, the results gathered from this study are in line with those of previous studies by Bankiqued et al [60] and Ángeles Quiroga et al [104]. The study by Bankiqued et al [60] found that casual games that tap working memory and reasoning can be robustly related to performance on working memory and fluid intelligence. Similar research on commercial video games by Ángeles Quiroga et al [104] found a strong relationship between performance in video games and general intelligence test performance. Our results confirm these findings at a finer granularity and show that when scrutinizing PAs, time-based, error-based, and result-based biomarkers yield promise in particular.

### Future Work

In this study, the participants with MCI were diagnosed with multiple-domain aMCI based on the diagnostic criteria described in the study by Petersen [95]. As MCI is a multidimensional clinical entity, it would be interesting to explore whether Klondike Solitaire game sessions are suitable for monitoring the cognitive status of participants with non-aMCI as well. The focus on executive functioning can be useful for identifying both MCI subtypes because it has been shown that both have a similar decrease in executive functioning [105]. Although we acknowledge that the evaluation of other cognitive functions such as anterograde memory, retrograde memory, orientation, and language is paramount to obtain a complete overview of the patient’s cognitive profile, these cognitive functions were not identified by the experts and were thus not included in our analysis.

### Reflections on the Use of COTS Games to Assess Cognitive Performance

COTS games also have their limitations. First, neuropsychological assessments are typically designed to assess a broad yet targeted spectrum of cognitive functions. Moreover, different tests are devised to measure 1 cognitive function in particular. COTS games, and more particularly digital card games, were found to be more limited in terms of the cognitive functions that they can specifically assess. When using COTS games, it may be hard to separate the evaluations of specific cognitive functions. In this study, experts judged every single PA to be moderately to strongly related to at least one cognitive function.

Second, using COTS games as an instrument to measure cognitive performance and possibly flag MCI necessitates ethical reflection. We envisioned that COTS games would be used only in accordance with the informed consent of the patient, with the positive aspiration that this could aid in the longitudinal monitoring of cognitive deterioration, more accurately measuring cognitive performance and variance. This project grew out of an ambition to escape the limitations of serious games and provide meaningful play to older adults. Nonetheless, we have to acknowledge that we may have transformed an activity previously considered enjoyable and innocent into an instrumental activity that may even trigger a sense of being under health surveillance [106]. Observational notes taken during this study did not reveal any verbal remarks of stress from the participants diagnosed with MCI. However, such remarks were made by some of the healthy participants because they felt pressure to outperform the participants living with MCI. Further research is needed to understand how the instrumentalization of COTS games affects the playing experience of patients.

Third, it has to be noted that deriving digital biomarkers from digital games may not be relevant for all older adults. Not everyone is an avid gamer, and even avid gamers may have preferences for different game genres. In addition, these preferences might change over time [14]. Although digital card games such as Klondike Solitaire are in general a popular pastime for the population susceptible to MCI [61,71,107-109], they might not be so for the coming generations. Therefore, it is important to identify other accessible games suitable for cognitive monitoring with a broad appeal.

Finally, the interaction between health care professional and patient, often stimulating and motivating in and of itself, is crucial for full assessment. Hence, we argue that COTS games for screening and monitoring of MCI should not be used as a replacement for current neuropsychological examination but rather as a source of additional information.

### Limitations

#### Fine-Tuning β Errors

In contrast with expectations, β error–related digital biomarkers proved to be insignificant. Upon inspecting the games of both groups, it became clear that there are two types of β errors: build stack β errors and suit stack β errors. The former represents missed moves among the build stacks. These errors were rarely made on purpose and occurred fewer times in the healthy participants’ group, based on observation. In contrast, the latter represent missed moves between build stacks and suit stacks. We observed that this latter category was used strategically to prevent the inability to place future cards. Our observations suggest that these occurred more often in the healthy participants’ group. However, because of the current configuration of the app, it was impossible to discriminate between these two types of β errors. Hence, this points to the importance of further contextualization and refinement of the measurement of β errors, and biomarkers in general, which should be addressed in future work.

#### Limited Sample Size

An a priori power analysis [110] estimated an adequate sample size to be between 32 and 88 participants (assuming comparable effect sizes as cognitive screening instruments to detect MCI [111]). Because of the strict inclusion criteria, only 46 participants were eligible. Although this strict protocol was designed with data quality in mind, the sample size may have affected the effects estimated in this study. It could be that our study was underpowered, leading to some digital biomarkers to be wrongly found insignificant. Future studies should therefore critically inspect the different digital biomarkers and the results obtained.

In addition, because of the average age difference between the 2 groups, we chose a GLMM for our statistical analysis because it can factor in confounding effects. A side exploration included using trained machine learning models on the same data set to predict age instead of MCI. These models were found to be less performant than the ones modeling MCI, underscoring that the effect of MCI was greater than the effect of age in our data set. Nevertheless, it is a limitation that we have to acknowledge and take into account while interpreting the results.

### Conclusions

This study provides insight into the cognitive functions addressed while playing digital card games and assesses the potential of digital card game sessions for screening for MCI. To this end, 11 experts in neuropsychology or geriatrics mapped the associations of PAs in Klondike Solitaire with cognitive functions. On the basis of this exercise, which showed that the experts agreed that PAs were related to cognitive functions, 23 potential digital biomarkers of cognitive performance were crafted. A GLMM analysis, taking the effects of age, tablet experience, and Klondike Solitaire experience into account, compared digital biomarker performance between a group consisting of people living with MCI and a healthy control group. We found that of the 23 digital biomarkers, 12 (52%) had a significant and sizeable effect, despite the strict inclusion criteria and natural variations in human cognition. These exploratory results support the notion of detecting MCI through Klondike Solitaire game sessions. 

## Appendix

Multimedia Appendix 1 Intraclass correlations of the cognitive functions and player actions with 95% CIs.

## Abbreviations

aMCI: amnestic mild cognitive impairment
CDR: Clinical Dementia Rating
COTS: commercial off-the-shelf
GLMM: generalized linear mixed model
ICC: intraclass correlation
MCI: mild cognitive impairment
MMSE: Mini-Mental State Examination
MoCA: Montreal Cognitive Assessment
naMCI: nonamnestic mild cognitive impairment
PA: player action
