# Supplementary material for: Dissecting Digital Card Games to Yield Digital Biomarkers for the Assessment of Mild Cognitive Impairment: Methodological Approach and Exploratory Study
Source: JMIR Serious Games. 2021 Nov 4;9(4):e18359. doi: 10.2196/18359 (PMC8603181; doi:10.2196/18359)
Supplement: Multimedia Appendix 1 [file games_v9i4e18359_app1.docx]

# Multimedia Appendix 1

| Variable of interest | Intraclass Correlation | 95% Confidence Interval | |
| --- | --- | --- | --- |
|  |  | Lower Bound | Upper Bound |
|  |  |  |  |
| Cognitive Function |  |  |  |
| Mental Flexibility | 0.68 | 0.43 | 0.85 |
| Inhibitory Control | 0.83 | 0.69 | 0.92 |
| Working memory | 0.82 | 0.67 | 0.91 |
| Selective Attention | 0.81 | 0.66 | 0.91 |
| Visuospatial Ability | 0.42 | -0.03 | 0.73 |
| Object Recognition | 0.66 | 0.39 | 0.84 |
| Apraxia | 0.71 | 0.48 | 0.86 |
| Cognitive Planning | 0.79 | 0.62 | 0.90 |
| Processing Speed | 0.87 | 0.78 | 0.94 |
|  |  |  |  |
| Player Action |  |  |  |
| PA1 | 0.92 | 0.81 | 0.98 |
| PA2 | 0.84 | 0.63 | 0.96 |
| PA3 | 0.87 | 0.70 | 0.97 |
| PA4 | 0.85 | 0.64 | 0.96 |
| PA5 | 0.90 | 0.76 | 0.97 |
| PA6 | 0.91 | 0.78 | 0.98 |
| PA7 | 0.91 | 0.78 | 0.97 |
| PA8 | 0.87 | 0.69 | 0.96 |
| PA9 | 0.88 | 0.71 | 0.97 |
| PA10 | 0.87 | 0.69 | 0.97 |
| PA11 | 0.87 | 0.70 | 0.97 |
| PA12 | 0.82 | 0.56 | 0.95 |
| PA13 | 0.94 | 0.85 | 0.98 |
| PA14 | 0.83 | 0.59 | 0.95 |
| PA15 | 0.90 | 0.75 | 0.97 |
| PA16 | 0.86 | 0.68 | 0.96 |
| PA17 | 0.91 | 0.78 | 0.97 |
| PA18 | 0.86 | 0.68 | 0.96 |
| PA19 | 0.91 | 0.78 | 0.98 |
| PA20 | 0.91 | 0.79 | 0.98 |
| PA21 | 0.93 | 0.84 | 0.98 |
